# A Mini-ISY100 Transposon Delivery System Effective in γ Proteobacteria

**DOI:** 10.3389/fmicb.2019.00280

**Published:** 2019-02-27

**Authors:** Emanuele Conte, Linda Mende, Ian Grainge, Sean D. Colloms

**Affiliations:** ^1^Institute of Molecular Cell and Systems Biology, University of Glasgow, Glasgow, United Kingdom; ^2^School of Environmental and Life Sciences, University of Newcastle, Newcastle, NSW, Australia

**Keywords:** ISY100, transposon mutagenesis, knock-out library, ΦC31 integrase, cytochrome *c*

## Abstract

Transposons are invaluable biological tools for the genetic manipulation of microorganisms. ISY100 from *Synechocystis* sp. PCC6803 is a member of the Tc1/*mariner*/IS*630* superfamily, and is characterized by high transposition efficiency and a strong preference for TA target sequences. In this paper, we describe the design and application of a mini-ISY100 suicide vector for the *in vivo* creation of stable random transposon insertion libraries. The system was successfully applied in seven species belonging to four different orders of γ proteobacteria. In all cases, delivery using conjugation consistently showed the highest transposition efficiency compared to chemical transformation or electroporation. We determined the frequency of transposon insertions in all the species and proved the utility of the system by identifying genes involved in colony coloration in *Shewanella oneidensis*. The ease and the efficiency of the protocol developed here allow the creation of complete knock-out libraries in an extensive range of host microorganisms in less than a week with no requirement for preparatory modification.

## Introduction

Transposon insertion mutagenesis is a powerful technique to interrogate the entire genome of an organism, especially when other genetic manipulation tools are not available (van Opijnen and Camilli, 2013). The applications of this approach include the definition of essential genes (Akerley et al., 1998; Glass et al., 2006; Freed et al., 2016), determination of the underlying genetic causes of specific phenotypes (Rollefson et al., 2009; Ding and Tan, 2017) and the creation of complete non-redundant knock-out libraries for genetic studies in less-known species (Gallagher et al., 2013). All these studies take advantage of the ability of transposons to create a large pool of mutants by inserting at random sites, ideally covering the entire genome.

Transposons can also be used to insert a genetic cargo in the chromosomal DNA at random locations (Ehrmann et al., 1997), or to generate portable regions of homology that can act as substrate for other recombination enzymes to generate deletions or duplications (Hughes, 2007).

The majority of the transposon mutagenesis protocols described in the literature rely on members of the DD[E/D] family of transposases (Nesmelova and Hackett, 2010), which catalyze transposition via a cut-and-paste mechanism. Among these, derivatives of Tn5, Tn7, Mu, Tn10, and Himar1 represent a common choice for the mutagenesis of prokaryotes (Choi and Kim, 2009; Picardeau, 2010), while at least ten transposons, including Sleeping Beauty, Himar1, and PiggyBac have been exploited for gene transfer applications in eukaryotes (Ni et al., 2008). The use of heterologous transposons has generally been preferred to avoid possible interference from related mobile elements in the host genome (Rubin et al., 1999). The complete lack of host requirements of *mariner* transposons of eukaryotic origin, like Himar1, allows their routine use in bacteria (Rubin et al., 1999), while in some cases transposons of bacterial origin (e.g., Tn5) have been used to mutagenize eukaryotic organisms with complete functionality (Suganuma et al., 2005).

Although lack of insertion site preference is desirable for mutagenesis experiments, all transposons display a certain degree of target site selectivity, and none of them can be truly defined as “random” (Liu et al., 2005). Target site preferences vary for different transposons and can be guided by requirement for a specific sequence (Wolkow et al., 1996), by the transcriptional status of a genomic region (Fraser et al., 1996) or by topological factors like the bending of the DNA in GC rich sequences (Green et al., 2012). These deviations from the assumed randomness can influence the result of an experiment, and limit coverage of the library (Kimura et al., 2016). The use of different transposons in the same species can lead to different and complementary results (Jacobs et al., 2003; Liberati et al., 2006). Furthermore, a particular transposon system could have a limited efficiency or not work at all in a species of interest (Bouhenni et al., 2005), or show an insertion site preference in a specific host (Bardarov et al., 1997), creating the need for an alternative method. It is, therefore, desirable to have multiple options to choose from. To expand the range of tools available for the genetic manipulation of different organisms, we set up a system relying on the use of the ISY100 (ISTcSA) transposon from *Synechocystis* sp. PCC6803 (Feng and Colloms, 2007).

ISY100 transposon is a member of the Tc1/*mariner*/IS630 family with a size of 947 bp and a single open reading frame coding for its own transposase (Urasaki et al., 2002). The transposase from ISY100 recognizes two 24 bp imperfect inverted repeats at the transposon ends, and has a marked preference for TA insertion sites (Feng and Colloms, 2007). ISY100 was isolated from the chromosome of *Synechocystis* sp. PCC6803, where it is present in 20 copies (Cassier-Chauvat et al., 1997; Urasaki et al., 2002). Its cut-and-paste reaction mechanism was studied both *in vivo* (Urasaki et al., 2002) and *in vitro* (Feng and Colloms, 2007), and showed a high transposition efficiency into the chromosome of *Escherichia coli* (Urasaki et al., 2002). To take advantage of these qualities, we designed a mini-ISY100 delivery system based on a R6K suicide vector and compared the efficiency of different delivery methods. Mini ISY100 transposition was tested in multiple strains of *E. coli* and in six other species of γ proteobacteria, producing insertion libraries that could be screened for mutant phenotypes.

## Materials and Methods

### Bacterial Strains

A list of the bacterial strains used in this study is shown in Table 1. All species were grown in LB medium (Sambrook and Russell, 2001), supplemented when necessary with 0.3 mM thymidine, 0.3 mM 2,6-diaminopimelic acid (DAP), chloramphenicol (25 μg/ml) or kanamycin (50 μg/ml for all; except for *Pseudomonas aeruginosa* 300 μg/ml). Solid medium was obtained by adding 15 g/l of agar. Bacteria were incubated at 37°C (*Escherichia coli, Acinetobacter baumannii, Acinetobacter baylyi*, and *Pseudomonas aeruginosa*) or 30°C (*Shewanella oneidensis, Pantoea ananatis* and *Pseudomonas fluorescens*).

**Table 1:** Bacterial strains used in this study.

Bacterial strain	Genotype	Source or reference
*E. coli* DH5α	(F^-^) *supE44* Δ(*lac*)U169 (φ*80lacZ*Δ*M15*) Δ(*argF*) *hsdR17 recA1 endA1 gyrA96 thi-1 relA1*	Taylor et al., 1993
*E. coli* TOP10	(F^-^) *mcrA* Δ*(mrr-hsdRMS-mcrBC)* φ*80lacZ*Δ*M15* Δ(*lac*)*X74 nupG recA1 araD139* Δ(*ara-leu*)*7697 galE15 galK16 rpsL*(*StrR*) *endA1* λ^-^	(Invitrogen) Presumed identical to DH10B (Grant et al., 1990)
*E. coli* MG1655	K-12 F^-^ λ^-^ *ilvG rfb-50 rph-1*	Bachmann, 1996
*E. coli*π3	TG1 (F^-^) Δ*thyA::(erm-pir116) [EmR]*	Demarre et al., 2005
*E. coli* MFD*pir*	MG1655 *RP4-2-Tc::*[Δ*Mu1::aac(3)IV*-Δ*aphA*-Δ*nic35-*Δ*Mu2::zeo*] Δ*dapA::*(*erm-pir*) Δ*recA*	Ferrières et al., 2010
*S. oneidensis* MR-1	Wild-type	Venkateswaran et al., 1999
*P. ananatis*	Wild-type	DSMZ collection No.30071
*P. fluorescens* SBW25	Wild-type	Rainey and Bailey, 1996
*A. baumannii* ATCC19606	Wild-type	Bouvet and Grimont, 1986
*A. baylyi* ADP1	Wild-type	Metzgar et al., 2004
*P. aeruginosa* PAO1	Wild-type	Holloway, 1975


### pISY100mini Plasmid Construction

The pISY100mini suicide plasmid was created by inserting a synthetic DNA sequence into the R6K γ origin plasmid pSW23T (Demarre et al., 2005) between the SalI and the SacI restriction sites. The inserted DNA sequence includes ISY100 inverted repeats IRL and IRR flanking the aminoglycoside phosphotransferase gene (kanamycin resistance, Kan^R^) from Tn5 (Beck et al., 1982), forming the mini-ISY100 transposon, followed by the T7 g10-L ribosome binding site (Olins and Rangwala, 1989) and the ISY100 transposase gene (Feng and Colloms, 2007). The pISY100mini-LP plasmid contained a ΦC31 integrase-based “landing pad” in pISY100mini, and was constructed by inserting synthetic DNA sequences containing a ΦC31 *attB*^TT^ site (GTGCCAGGGCGTGCCCTTGGGCTCCCCGGGCGCG) and the counter-selectable *E. coli rpsL* gene between the AatII and BsaI sites of pISY100mini, and a fragment containing an *attB*^TC^ site (GTGCCAGGGCGTGCCCTCGGGCTCCCCGGGCGCG), in the opposite orientation, between the NheI and the SpeI sites. Replication of these ISY100 donor plasmids requires the *pir* gene, therefore both plasmids were maintained in *E. coli* π3 (Demarre et al., 2005). All plasmids were verified by DNA sequencing.

### Chemical Transformation and Electroporation

Suicide plasmids were introduced into bacteria using chemical transformation or electroporation. Chemical transformation of *E. coli, P. ananatis, P. fluorescens, S. oneidensis* used a standard heat-shock protocol with calcium chloride treated cells (Sambrook and Russell, 2001). To prepare electro-competent cells, 200 ml of exponentially growing culture (OD600 = 0.4) was centrifuged for 7 min at 8000 g then resuspended in 100 ml pre-chilled 10% glycerol. Centrifugation was repeated three more times with cells resuspended first in 50 ml and then in 25 ml of 10% glycerol, before final resuspension in 500 μl of 10% glycerol. Electroporation was performed in 0.2 cm cuvettes at 2.5 kV (0.55 kV for *S. oneidensis*) using 40 μl of cells mixed with 165 ng of plasmid DNA. After electroporation, 1 ml of LB (with thymidine for π3) was added and cells were incubated with shaking for 1.5 h at 37°C or 30°C as appropriate. After this, an aliquot of cells was spread on selective plates. All the experiments were repeated in triplicate, and results reported as mean ± standard deviation.

### Conjugation

The donor strain for the conjugation experiments was obtained by introducing pISY100mini or pISY100mini-LP into *E. coli* MFD*pir* (Ferrières et al., 2010) by chemical transformation. Overnight cultures of donor and recipient strains (1 ml each) were pelleted by centrifugation (3000 g – 5 min) and washed twice in 500 μl of dilution buffer (10 mM Tris pH 7.5, 10 mM MgSO_4_, 68 mM NaCl_2_) before final resuspension in 500 μl of dilution buffer. The donor strain (100 μl) was mixed gently with 50 μl of the recipient cells (100 μl for *E. coli*) by pipetting up and down. The mixture was pipetted without spreading at the center of a LB/DAP agar plate and incubated at 30°C for 5 h (*A. baylyi, A. baumannii*, and *P. aeruginosa*), 8 h (*E. coli* and *S. oneidensis*) or overnight (16 h; *P. ananatis, P. fluorescens*). Cells were washed off the plate with 3 ml of LB (700 μl for the experiments conducted with pISY100-LP-mini) and serial dilutions were spread on plates containing kanamycin and no DAP, and incubated at 30 or 37°C for 48 h to select for recipient cells containing a mini-ISY100 transposon inserted into the chromosome. Each experiment was repeated independently three times to determine the mean and standard deviation.

### Arbitrary PCR

The protocol for colony arbitrary PCR was adapted from Das et al. (2005) using the primers KanR_3 GACCGCTTCCTCGTGCTTTAC, KanR_4 TCTATCGCCTTCTTGACGAGTTC, Arb1 GGCCACGCGTCGACTAGTCANNNNNNNNNNGATAT, and Arb2 GGCCACGCGTCGACTAGTCA. A single colony was picked from a plate, resuspended in 50 μl of distilled water and heated at 95°C for 15 min. An aliquot of this suspension (5 μl) was added to a PCR mixture containing 25 μl Taq 2X Master Mix (New England BioLabs), 2.5 μl each of primer Kanr-3 (10 μM) and ARB1-1 (50 μM) and 15 μl dH_2_O. This was subjected to an initial denaturation at 95°C for 5 min, followed by 6 cycles of 95°C – 30 s, 30°C – 30 s, 72°C – 1.5 min, and then 30 cycles of 95°C – 30 s, 45°C – 30 s, 72°C – 2 min, with a final elongation at 72°C for 4 min. Five microliter of this PCR product was used as DNA template in a second PCR mixture containing 25 μl Taq 2X Master Mix (New England BioLabs), 2.5 μl each of primer Kanr-4 and Arb2 (10 μM each) and 15 μl dH_2_O and subjected to the following program: initial denaturation 95°C – 5 min, then 30 cylcles of 95°C – 30 s, 52°C – 30 s, 72°C – 2 min, followed by 72°C – 4 min. The PCR products were purified using a Qiagen PCR cleanup kit and 15 μl of each reaction was sequenced with primer Kanr-4.

### Estimation of the Mutant Library Coverage

The completeness of a transposon insertion mutant library can be predicted by assuming a Poisson distribution for the frequency of the transposon insertions in the genome (Baym et al., 2016). Essential genes will not be represented in the library, and are excluded from the calculations. Assuming a genome with N non-essential genes, all with an equal probability of insertion, any single gene will be targeted by the transposase with a probability of:

$$P\left(x,k,N\right)=e^{-k/N}{\left(\frac{k}{N}\right)}^{x}\left(\frac{1}{x!}\right)$$

Where x represents the number of insertions in the gene and k the number of mutants in the collection. From this, the probability of no insertion in any given gene is:

$$P\left(0,k,N\right)=e^{-k/N}$$

Meaning that the total number of genes that will not have any insertions is:

$$n_{no\;insertions}=Ne^{-k/N}$$

By complementarity, the total number of genes that will have at least one transposon insertion is:

$$n_{insertions}=N\left(1-e^{-k/N}\right)$$

To estimate the number of essential genes, we referred to the empirically determined relationship of Gao et al. (2015). According to this, the percentage of essential genes y in a bacterium decays exponentially in relation to the length x of the genome following the equation:

$$y=144e^{-x/719649}+12$$

The information on the size of the genome and the number of genes for the analyzed bacteria was derived from the KEGG database^1^.

## Results

### Plasmid Design

**FIGURE 1:**
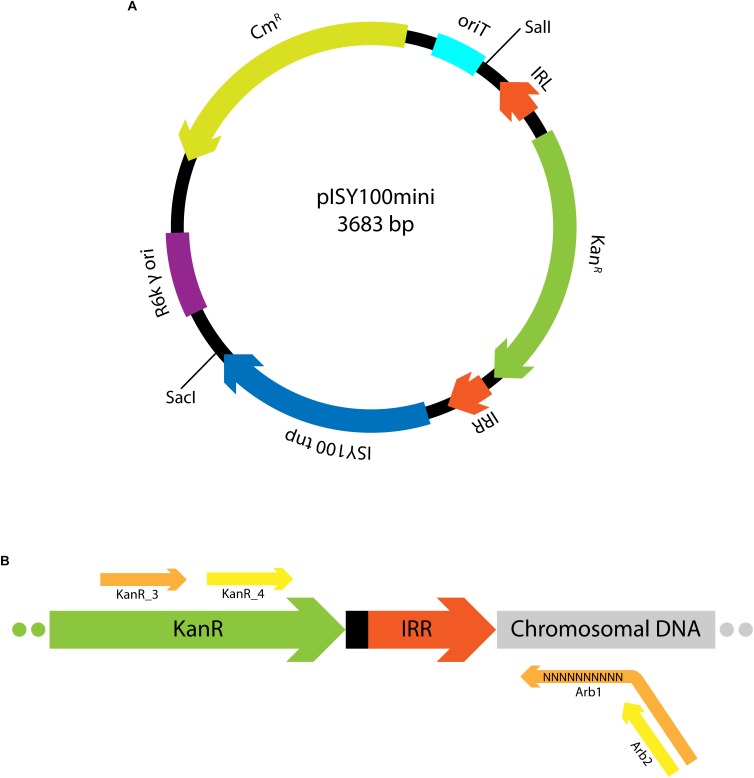
Mini-ISY100 delivery system. **(A)** Map of the pISY100mini suicide vector, including the R6K γ origin of replication (purple), the origin of transfer *oriT* (light blue), the chloramphenicol resistance gene (yellow), the ISY100 transposase gene (blue), the ISY100 inverted repeat sequences IRL and IRR (dark orange) and the kanamycin resistance gene (green). **(B)** mini-ISY100 inserted in the host chromosome. The primers used in the arbitrary PCR to identify the mini-ISY100 transposon insertion site in the chromosomal DNA (light gray) are represented in orange (KanR_3 and Arb1, first amplification step) or yellow (KanR_4 and Arb2, second amplification step).

The aim of this work was to design and test a suicide plasmid for the delivery of ISY100 mini-transposon insertions to the genomes of a variety of bacterial species. The suicide plasmid (pISY100mini; Figure 1A) contains a mini transposon (mini-ISY100), consisting of the Tn5 aminoglycoside phosphotransferase gene (Kan^R^) between ISY100 inverted repeat sequences (IRL and IRR), and has an R6K γ origin of replication. Replication of pISY100mini is strictly dependent on the R6K *pir*-encoded Π protein, which is not encoded on the plasmid but can be provided *in trans* allowing replication in *pir*^+^ host strains. ISY100 transposase is encoded on pISY100mini outside the mini-ISY100 transposon. After introducing pISY100mini into a *pir*^-^ recipient strain, the plasmid cannot replicate, and kanamycin resistant colonies will arise only if the mini-ISY100 transposes to the chromosome. Insertions should be stable as the transposase gene is left behind on the non-replicating donor plasmid. pISY100mini carries a gene conferring resistance to chloramphenicol (Cm^R^) outside the mini-ISY100 so that colonies arising from transposition should be kanamycin resistant but chloramphenicol sensitive.

The suicide plasmid could be delivered to recipient cells by transformation, however, conjugation might be a more efficient delivery system. An IncP origin of transfer (*oriT*) was therefore included on the pISY100mini backbone to allow mobilization of pISY100mini using the RP4 conjugative machinery.

### Transposition in *E. coli*

To test the function and the efficiency of our mini-ISY100 delivery system, transposition was assayed in two commonly used (*pir*^-^) strains of *E. coli*: DH5α and TOP10. The pISY100mini suicide plasmid was introduced into these strains using a simple chemical transformation protocol. No colonies were obtained when samples were spread on plates containing chloramphenicol, confirming that the suicide plasmid cannot replicate in these host strains. The number of cells in which transposition had taken place was determined by counting the number of colonies on LB plates containing kanamycin. This number ranged from an average of 18 per transformation for DH5α, to about 120 for TOP10 (Table 2). The total number of cells surviving the transposition procedure was determined by plating on non-selective plates, and the transposition frequency was then calculated by dividing the number of kanamycin resistant colonies by the total number of colony forming units (Table 2). The results show that approximately 1 in 800,000 (DH5α) to 1 in 280,000 (TOP10) cells in the transformation undergo transposition onto their chromosome.

**Table 2:** Delivery of the mini-ISY100 transposon in *E. coli* via chemical transformation and electroporation.

	pISY100mini		pUC71K (control)	
			
Strain	Kan^R^ colonies/reaction^a^	Transposition frequency^b^	Transformation frequency^b^	Mini-ISY100 activity^c^
**Chemical transformation**				
DH5α	17.5 ± 3.54	1.29 × 10^-6^± 2.95 × 10^-7^	1.40 × 10^-3^± 2.71 × 10^-4^	9.21 × 10^-4^± 2.76 × 10^-4^
TOP10	117 ± 28.9	3.57 × 10^-6^ ± 9.47 × 10^-7^	7.32 × 10^-3^ ± 7.43 × 10^-4^	4.88 × 10^-4^ ± 1.39 × 10^-4^
**Electroporation**				
DH5α	1730 ± 297	3.95 × 10^-6^ ± 1.28 × 10^-6^	5.49 × 10^-2^ ± 6.45 × 10^-3^	7.19 × 10^-5^ ± 2.50 × 10^-5^
TOP10	2280 ± 254	1.10 × 10^-5^ ± 2.14 × 10^-6^	7.03 × 10^-2^ ± 1.72 × 10^-2^	1.56 × 10^-4^ ± 4.89 × 10^-5^


*^*a*^Number of Kan^*R*^ colonies obtained from a single reaction. ^*b*^Number of colonies on LB/Kan divided by the number of colonies on LB. ^*c*^Transposition frequency divided by transformation frequency.*

Production of a transposon insertion by transformation with pISY100mini requires two successful steps: delivery of DNA into the cell, followed by transposition from the suicide plasmid to the host genome. To separate these two different events, we defined the transposition “activity” (Table 2) as the ratio between the transposition frequency of pISY100mini, depending on both transposition and DNA uptake, and the transformation frequency of the cells which depends just on delivery of the DNA. The transformation efficiency cannot be measured in DH5α and TOP10 using pISY100mini, as this plasmid cannot replicate in these cells. Instead, the efficiency of DNA delivery by chemical transformation was measured for each batch of competent cells, using the plasmid pUC71K (Taylor and Rose, 1988). This plasmid is comparable in size to pISY100mini and carries the same Kan^R^ gene, but can be maintained in a *pir^-^* host due to its pMB1/ColE1 origin of replication. The results indicate that after chemical transformation, in the order of 1 in 1000 cells takes up plasmid DNA, and of these about 1 in 1000 has a transposition event to the chromosome (Table 2).

To increase the transformation frequency, electroporation was then tested to deliver pISY100mini for transposition. In these experiments, the transformation frequency increased, but the transposition activity decreased, so that the overall transposition frequency was similar to that obtained with chemical transformation (Table 2). Because of the large number of cells used in the electroporation reactions, significantly more transposition events were obtained by electroporation than by chemical transformation; ∼2000 colonies were obtained by electroporation with both DH5α and TOP10 (Table 2). Thus, a library large enough to have nearly full coverage of all non-essential genes in *E. coli* (requiring ∼12,000 insertions mutants) could be produced by combining colonies from several electroporation reactions.

Conjugation is a highly efficient method of delivering plasmid DNA to bacterial cells, so we tested it as an alternative delivery method for pISY100mini. The suicide plasmid pISY100mini was introduced into *E. coli* MFD*pir* (Ferrières et al., 2010) by chemical transformation. This strain expresses the *pir* gene for replication of pISY100mini and the RP4 conjugative proteins, allowing mobilization of pISY100mini. MFD*pir* also contains a mutation in the *dapA* gene, allowing easy counterselection after conjugation using media lacking diaminopimelic acid (DAP).

A plate-based mating reaction was set up between the MFD*pir* pISY100mini donor strain and the two different recipient strains, DH5α and TOP10, as described in the Materials and Methods section. After 8 h of co-incubation of donor and recipient strains on LB DAP plates, cells were washed off and dilutions were plated on different media. No colonies were obtained on LB Cm plates, confirming that pISY100mini cannot replicate in the recipients, and that there is no reversion of the *dapA* mutation in the donor. LB plates lacking DAP were used to count the total number of recipient cells, while LB lacking DAP but containing kanamycin was used to select for transposition events in DH5α or TOP10. Libraries of 10^5^ transposon insertion mutants were routinely obtained from each conjugation reaction (Table 3), although it should be noted that due to the extended incubation times during the conjugation, these colonies probably do not all represent independent transposition events. The transposition frequency after conjugation, ∼10^-2^–10^-3^, calculated as the proportion of recipient cells that became kanamycin resistant, was significantly higher than obtained by transformation, ∼10^-5^–10^-6^ (compare Tables 2, 3).

**Table 3:** mini-ISY100 transposon activity in *E. coli* after conjugation.

Strain	Kan^R^ colonies/reaction^a^	Transposition frequency^b^	Mini-ISY100 activity^c^
DH5α	1.05 × 10^5^ ± 2.12 × 10^4^	1.54 × 10^-2^ ± 8.25 × 10^-3^	4.20 × 10^-1^ ± 3.70 × 10^-1^
TOP10	3.50 × 10^5^ ± 1.71 × 10^5^	1.75 × 10^-3^ ± 9.71 × 10^-4^	4.78 × 10^-2^ ± 4.26 × 10^-2^


*^*a*^Number of Kan^*R*^ colonies obtained from a single reaction. ^*b*^Number of colonies on LB/Kan divided by the number of colonies on LB. ^*c*^Transposition frequency divided by frequency of conjugation using E. coli π3 as recipient (3.66 × 10^-*2*^ ± 2.55 × 10^-*2*^).*

The conjugation frequency of pISY100mini from MFD*pir* was measured by mating MFD*pir*/pISY100mini with the *pir*^+^ *E. coli* strain π3, allowing replication of pISY100mini in the recipient. Cells were plated on LB, to count the total number of recipient cells, and LB/Kan+Cm, to determine the number of transconjugants, giving a conjugation frequency of ∼4 × 10^-2^. The transposition activity of the mini-ISY100 transposon after conjugation in the *E. coli pir^-^* strains was then estimated by dividing the transposition frequency by the conjugation frequency of pISY100mini from MFD*pir* to π3 (Table 3). These calculations indicate that a substantial proportion of cells that receive pISY100mini by conjugation (as high as 40% for DH5α) undergo transposition from pISY100mini to the host chromosome.

### Creation of Knock-Out Libraries in γ Proteobacteria

To show that pISY100mini can be used to create transposon insertion libraries in other bacterial species, we tested transposition in *S. oneidensis, P. ananatis*, and *P. fluorescens*. These species have all shown biotechnological potential (Fredrickson et al., 2008; Silby et al., 2009; Hara et al., 2012), and are representatives of three different orders of γ proteobacteria. Our first attempts to deliver pISY100mini in these species using chemical transformation and electroporation were unsuccessful, producing no Kan^R^ colonies. Therefore, we attempted to deliver pISY100mini by inter-species conjugation, using *E. coli* MFD*pir*/pISY100mini as donor. Conjugation was conducted on LB DAP plates by mixing donor and recipient strains as described in the Materials and Methods section, and leaving them to incubate at 30°C for 8–16 h. No colonies were obtained when the conjugations were plated on LB Cm plates, demonstrating that pISY100mini cannot replicate in any of these species, and controls with individual parental strains produced no colonies on LB Kan plates. Conjugation between MFD*pir*/pISY100mini and the different recipient species yielded from 10^3^ to 10^5^ Kan^R^ colonies, depending on the recipient species. Transposition frequencies (the fraction of recipient cells that had become Kan^R^) were in the order of 10^-6^ for *P. fluorescens*, 10^-5^ for *P. ananatis* and 10^-4^ for *S. oneidensis* (Table 4).

**Table 4:** mini-ISY100 transposition frequency in different species.

Recipient strain	Kan^R^ colonies/reaction^a^	Transposition frequency^b^
*S. oneidensis*	1.94 × 10^5^ ± 2.38 × 10^4^	2.48 × 10^-4^± 1.42 × 10^-4^
*P. ananatis*	2.18 × 10^4^ ± 1.87 × 10^3^	1.04 × 10^-5^ ± 1.4 × 10^-6^
*P. fluorescens*	7.04 × 10^3^ ± 3.80 × 10^2^	1.80 × 10^-6^ ± 6.14 × 10^-7^


*^*a*^Total number of Kan^*R*^ colonies obtained from a single plate mating between MFDpir and the indicated recipient bacteria, calculated from plating appropriate dilutions. ^*b*^Number of colonies on LB/Kan divided by the number of colonies on LB.*

To confirm that the observed Kan^R^ colonies were due to mini-ISY100 transposon insertions in the recipient host genome, arbitrary PCR was used to amplify the DNA flanking transposon insertion sites (Das et al., 2005). Five Kan^R^ colonies produced by conjugation of MFD*pir*/pISY100mini with each species were randomly selected and subjected to colony PCR using a forward primer specific for the kanamycin resistance gene and a reverse “arbitrary” primer containing a 10 bp random sequence at its 3′ end. The products of this reaction were further amplified by PCR using a nested primer from the kanamycin resistance gene and a reverse primer matching a fixed region from the 5′end of the degenerate primer (Figure 1B). PCR products were sequenced by dideoxy sequencing using a primer from the kanamycin resistance gene, allowing unique determination of the mini-ISY100 transposon insertion site (Supplementary Table S1). All the transposon insertions could be identified in the genomic sequence of the target organism and were at TA dinucleotides, as expected for ISY100.

To create a useful insertional knock-out library in the species studied, the library should be large enough for nearly full coverage of the genome. A practical target to aim for is for >95% of non-essential genes to have at least one insertion. From the Poisson distribution (see the Materials and Methods section), this requires approximately 3N insertions, where N is the number of non-essential genes in the genome. The number of non-essential genes has been determined for *S. oneidensis* as N_oneidensis_≅3700 genes (Baym et al., 2016), and can be estimated from the annotated genome sequences of *P. ananatis* (Hara et al., 2012) and *P. fluorescens* (Silby et al., 2009). *P. ananatis* has a genome of 4.88 Mbp, with 4067 protein-coding genes, while *P. fluorescens* has a genome of 6.72 Mbp, with 5921 protein-coding genes. Based on their genome sizes, about 12% of these genes are expected to be essential (Gao et al., 2015) resulting in N_ananatis_≅3600 and N_fluorescens_≅5200. Thus, libraries ranging from 12,000 to 16,000 mutants are required for 95% genomic coverage of *S. oneidensis, P. ananatis* and *P. fluorescens.* Therefore, the numbers of mini-ISY100 transposition events obtained with our delivery system are sufficient to create knock-out libraries with 95% coverage in *S. oneidensis* and *P. ananatis* in a single experiment, while two independent conjugation reactions of this scale would be required for *P. fluorescens* (Table 4).

### Identification of Genes Involved in Colony Color in *Shewanella oneidensis*

**FIGURE 2:**
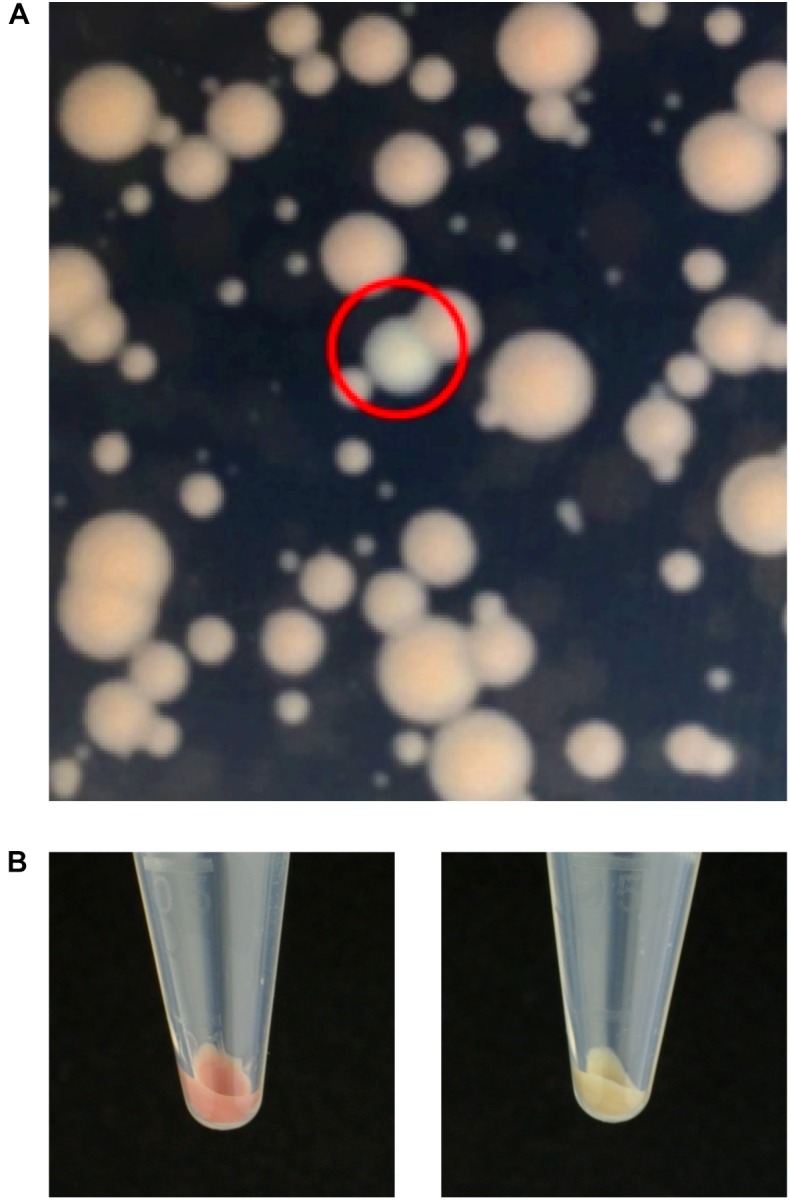
Genetic screen for genes involved in *S. oneidensis* colony color. A mini-ISY100 insertion library was generated and plated on LB/Kan to screen for mutants that had lost the red-brown coloration. **(A)** Close-up of kanamycin resistant *S. oneidensis* colonies obtained after conjugation with MFD*pir*/pISY100mini. Heterogeneity in colony size might be due to fitness differences caused by transposon insertions. Alternatively, small colonies might reflect late (post-plating) transposition events. A mutant with pale colony color is circled in red. **(B)** Pellets obtained after centrifuging 2.8 ml LB liquid cultures (30°C, overnight) showing the difference in color between the wild-type *S. oneidensis* (left) and a pale mutant (right).

Having proved the efficiency of transposition using pISY100mini, we wished to demonstrate the utility of ISY100 for genetic screens. *S. oneidensis* produces colonies with a pale brown coloration on LB agar plates and we decided to screen for mutations in genes required for this colony color. A library of *S. oneidensis* mutants was created by mating MFD*pir*/pISY100mini with *S. oneidensis* and selecting on LB Kan plates. From a library of approximately 12,000 mutants, four white colonies were identified (Figure 2) and streaked to single colonies. Arbitrary PCR was used to amplify the DNA flanking the mini-ISY100 insertions in these mutants, and the insertion site was determined by DNA sequencing. Three of the mutants had insertions in the *ccmF* gene of *S. oneidensis*, at three different locations within this gene, while the fourth colony had an insertion in the *dsbD* gene (Table 5). The product of *ccmF*, a cytochrome *c* maturation protein, has a role in the reduction of heme for its transfer to the apocytochrome *c* (Verissimo and Daldal, 2014). The *dsbD* gene encodes a membrane protein belonging to the thioredoxin family, whose function in bacteria is to maintain apocytochrome *c* in a reduced form by acting on the assembly protein CcmG (Verissimo and Daldal, 2014). Both of these proteins are therefore involved in the biogenesis and maturation of cytochrome *c*, indicating that a defect in this pathway might underlie the observed pale colony color phenotype. Indeed, a pale colony phenotype has previously been observed for mutants in *ccmF* in *S. oneidensis* (Fu et al., 2015). A causal relationship between the mini-ISY100 insertion in *dsbD* and pale colony color should be confirmed by complementation and/or gene knockout experiments.

**Table 5:** Genome insertion sites of the mini-ISY100 in *S. oneidensis* pale colored colonies.

Mutant ID	Sequence^a^	Locus tag	Gene product	Genome insertion site
***Shewanella oneidensis* – NCBI: NC_004347.2**				
So_pale-1	(mini-ISY100)**TA**CTAATCGGATCGCC (plus/minus)	SO_0266	Cytochrome c-type biogenesis protein CcmF	267,474
So_ pale-2	(mini-ISY100)**TA**AATGAAGCGTTTTC (plus/minus)	SO_0266	Cytochrome c-type biogenesis protein CcmF	266,418
So_ pale-3	(mini-ISY100)**TA**ATACGCCCACCAAG (plus/minus)	SO_0266	Cytochrome c-type biogenesis protein CcmF	266,362
So_ pale-4	(mini-ISY100)**TA**TGGGCATGGGCGTG (plus/minus)	SO_0696	Protein-disulfide reductase DsbD	714,906


*^*a*^The orientation of the transposon relative to the genomic sequence is indicated in brackets (plus/plus indicates that the coding strand of the kanR gene is in the same orientation as the reported genome sequence; plus/minus indicates they are in opposite directions).*

### Delivery of a Genetic Cargo

**FIGURE 3:**
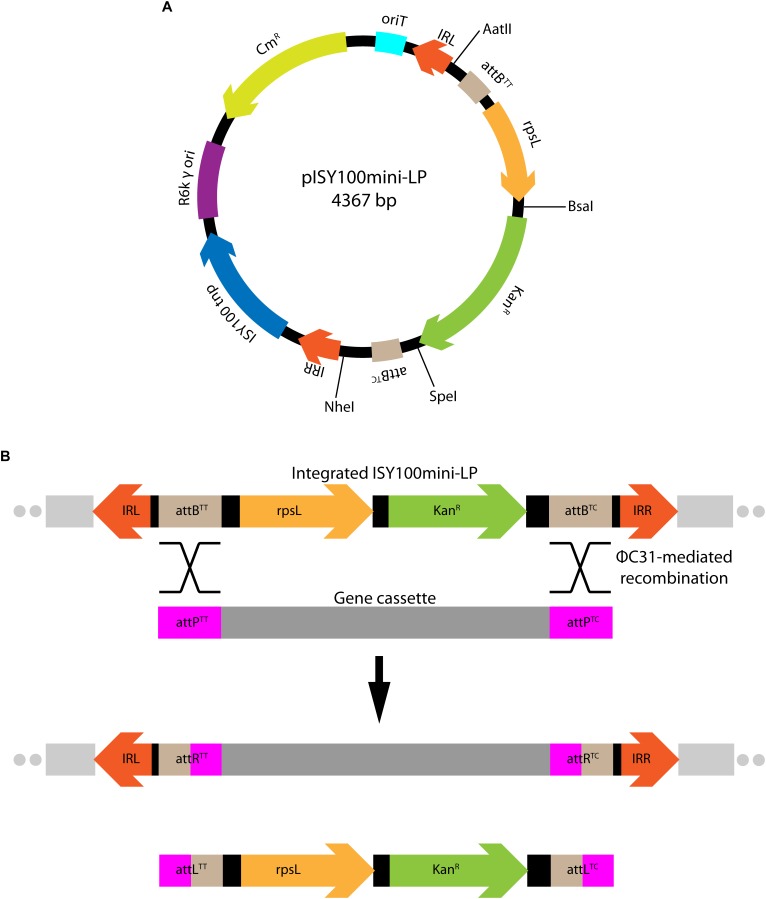
Delivery of a chromosomal “landing pad.” **(A)** Map of the pISY100mini-LP vector, designed to deliver a “landing pad” for ΦC31 integrase-mediated cassette exchange. The same color scheme as in Figure 1 was used for the plasmid backbone. Two ΦC31 *attB* sites (light-brown) and the *rpsL* gene (*E. coli* 30S ribosomal subunit protein S12 – light-orange) conferring streptomycin sensitivity to an otherwise streptomycin resistant *E. coli rpsL* mutant strain, were added in the mini-ISY100 transposon sequence. **(B)** Mini-ISY100-LP integrated on the chromosome and genetic cassette exchange *attB*/*attP* recombination. The two *attB* sites inserted in the landing pad can be used to integrate a gene cassette (dark gray) flanked by two *attP* sites (pink) via ΦC31 integrase-mediated recombination.

Next, we investigated the use of mini-ISY100 to deliver a genetic cargo to the genomes of different bacterial species. The sequence of pISY100mini was modified so that it could be used to deliver a chromosomal “landing pad” for ΦC31 integrase-mediated cassette exchange (Fogg et al., 2014). ΦC31 integrase catalyzes efficient unidirectional recombination between short DNA sites knows as *attP* and *attB* (Thorpe and Smith, 1998). A ΦC31 *attB* site with a TT central dinucleotide (*attB^TT^*) together with the *rpsL* streptomycin sensitivity gene from *E. coli* was placed at one end of the mini-ISY100 just inside the left inverted repeat, and *attB* with a TC central dinucleotide (*attB^TC^*) was placed at the other end, just inside IRR, creating pISY100mini-LP (Figure 3A). These changes add 684 bp to mini-ISY100, bringing the total size of the genetic cargo carried by mini-ISY100-LP to 1,679 bp. pISY100mini-LP can be delivered by conjugation as before, and transposon insertions can be selected using the kanamycin resistance gene. Once on the chromosome of a bacterial host, a genetic cassette flanked by *attP^TT^* and *attP^TC^* recombination sites could be integrated into the landing pad using ΦC31 integrase-mediated recombination (Figure 3B; S. D. Colloms, unpublished results). This cassette exchange could be selected for by loss of the *rpsL* (Reyrat et al., 1998) or gain of a marker carried on the cassette.

The ability of this larger landing pad ISY100 to transpose was tested in the following bacteria: *E. coli* MG1655, *A. baumannii* ATCC19606, *A. baylyi* ADP1 and *P. aeruginosa* PAO1. MG1655 was used as a wild-type *E. coli* control. *P. aeruginosa* and *A. baumannii* are human pathogens of high clinical importance – both are listed within the top three species where urgent need for additional research is required (World Health Organization, 2017). *A. baylyi* is a genetically tractable relative of *A. baumannii*. New genetic tools that work in these species could help identify genes required for their virulence and/or new drug targets.

MFD*pir*/pISY100mini-LP was used as a donor for conjugation. Conjugations were plated on LB to count recipient cells, LB Cm to verify that the donor plasmid cannot replicate in the recipient strains, and LB Kan to determine the number of transposition events. No colonies were obtained on LB Cm plates, whereas hundreds (*A. baumannii*) to over a million (*P. aeruginosa*) kanamycin resistant colonies were obtained from the conjugations (Table 6). Using arbitrary PCR and DNA sequencing, the locations of transposon insertions were determined in three random kanamycin resistant colonies for each species. Insertions were in apparently random TA dinucleotides in the genomes of all species (Supplementary Table S2).

**Table 6:** mini-ISY100-LP transposition frequency in different species.

Strain	Kan^R^ colonies/reaction^a^	Transposition frequency^b^
*Escherichia coli* MG1655	4.2 × 10^4^	4.1 × 10^-5^ ± 9.20 × 10^-6^
*Acinetobacter baumannii*	8.75 × 10^2^	1.2 × 10^-6^ ± 6.4 × 10^-8^
*Acinetobacter baylyi*	1.4 × 10^5^	9.8 × 10^-5^ ± 3. 0 × 10^-5^
*Pseudomonas aeruginosa*	1.05 × 10^6^	1.5 × 10^-3^


*^*a*^Total number of Kan^*R*^ colonies obtained from a single plate mating between MFDpir and the indicated recipient bacteria, calculated from plating appropriate dilutions. ^*b*^Number of colonies on LB/Kan divided by the number of colonies on LB.*

## Discussion

The use of transposons as tools for genetic engineering has recently undergone a large expansion. Here we describe the design and application of a new mini transposon insertion mutagenesis tool based on the ISY100 (ISTcSA) transposon from *Synechocystis* sp. PCC6803. In comparison with other members of the same *mariner* family, the advantages of the ISTcSA transposase are its small size (only 282 amino acids) and the possibility to be modified to specifically target precise sequences without a loss of functionality (Feng et al., 2010).

**FIGURE 4:**
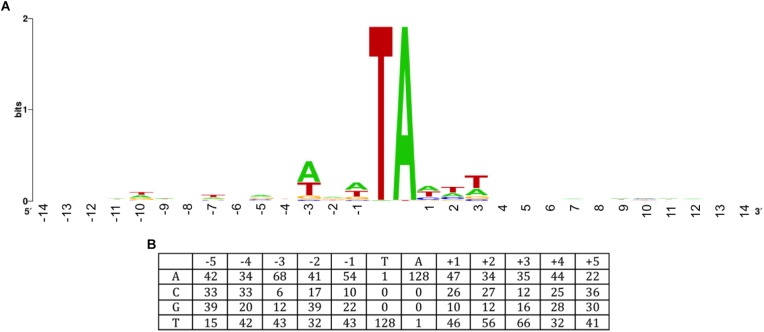
ISY100 target site preference. The sequences of 21 ISY100 insertions in the *Synechocystis* PCC6803 genome, three in large *Synechocystis* plasmids, 19 insertions isolated in plasmid targets in *E. coli*, and 58 different insertion sites generated by *in vivo* and *in vitro* transposition into the target plasmid pH2 were previously analyzed (Feng et al., 2010). The 28 insertions isolated in this work were added to this alignment and analyzed for conserved sequence features. **(A)** WebLogo (Crooks et al., 2004) showing sequence information content 14 nucleotides on either side of the target TA. **(B)** Base frequencies 5 nucleotides on either side of the target TA.

Like other members of the Tc1/*mariner* family, including Sleeping Beauty and Himar1, ISY100 inserts almost exclusively into TA dinucleotides. To see if there is any target site preference outside this TA, the sequences of 101 previously reported insertions sites (Feng et al., 2010) were combined with the 28 new insertion sites reported in this study (Figure 4). Analysis of these sequences suggests that there is a slight preference for insertion sites that are A/T-rich in the three nucleotides on either side of the TA target. This specificity differs from other transposases commonly used in prokaryotes, such as Mu {5′ CPy (G/C)PuG} or Tn5 {5′ GPyPyPy(A/T)PuPuPuC} (Green et al., 2012), but might be similar to other *mariner* transposons such as Sleeping Beauty (5′ ATATATAT) (Liu et al., 2005) and Himar1, where no consensus target sequence has been detected but sites with the sequence (C/G)GNTANC(C/G) have been found to be non-permissive for insertion (DeJesus et al., 2017).

The frequency of the mini-ISY100 transposition was shown to be affected by the method used to deliver the pISY100mini suicide plasmid to recipient cells. Conjugation was found to result in the most efficient transposition for all species tested and is thus our method of choice, as previously shown for other transposons (Christie-Oleza et al., 2009). Conjugation also represents the best technique to overcome the barriers to genetic exchange between different species, expanding the adaptability of our pISY100mini vector delivery system. Using the conjugation delivery system, we were able to obtain large libraries of knock-out mutants in seven different species of γ proteobacteria, belonging to four different orders. These results show the lack of requirement of any specific co-factor for the transposition of the mini-ISY100 transposon and open the possibility of a successful application of the delivery system in other species.

We applied our protocol to create and characterize a mini-ISY100 knock-out library in *S. oneidensis*, screening the mutants for loss of colony color. The analysis of the mini-ISY100 insertion sites in the chromosome of the pale colored *S. oneidensis* colonies led to the putative identification of two genes involved in this phenotype, *ccmF* and *dsbD*. While mutations in *ccmF* were previously associated with the appearance of pale colonies on LB media (Fu et al., 2015), this is the first time such a phenotype is reported for a mutation in *dsbD*, proving the utility of our system. The fact that four, rare, pale colored colonies from a library of ∼12,000 mutants all had different insertion sites, is convincing evidence that a large proportion of colonies in this *S. oneidensis* library were not siblings but came from independent insertion events.

We also created a modified version of the mini-ISY100 transposon, named mini-ISY100-LP, designed to deliver a landing pad in a random location of the host chromosome to allow the insertion of genetic cassettes mediated by the integrase from bacteriophage ΦC31. While the transposition frequency of the mini-ISY100-LP transposon in *E. coli* is reduced in comparison with the mini-ISY100 transposon, probably due to the presence of a larger genetic cargo, the efficiency of the system still yields large libraries of transposon insertions in all four species tested. We have yet to determine if there is an upper limit to the size of the genetic cargo.

The addition of ISY100 to our microbial genetic tool box complements existing transposon delivery systems and might facilitate development of new technologies that could for instance require orthogonal transposons, or different sequences in the inverted repeats. The high efficiency and the flexibility of pISY100mini should make it a valuable tool for genetic studies in a broad range of bacterial species.

## Author Contributions

EC, IG, and SC conceived and designed the work. EC, LM, and IG performed the experiments. EC drafted the manuscript. IG and SC critically revised the manuscript. All authors read and approved the final version of the article.

## Conflict of Interest Statement

The authors declare that the research was conducted in the absence of any commercial or financial relationships that could be construed as a potential conflict of interest.

## Footnotes

1 http://www.genome.jp/kegg/kegg2.html
