# Supplementary material for: A Mini-ISY100 Transposon Delivery System Effective in γ Proteobacteria
Source: Front Microbiol. 2019 Feb 27;10:280. doi: 10.3389/fmicb.2019.00280 (PMC6400869; doi:10.3389/fmicb.2019.00280)
Supplement: Supplementary file 1 [file Table_1.docx]

Supplementary Table S1 **– Genome insertion sites of the miniISY100 transposon**

| *Shewanella oneidensis* - NCBI: NC_004347.2 | | | | | | |
| --- | --- | --- | --- | --- | --- | --- |
| Mutant ID | Sequence | | Locus tag | | Gene product | Genome insertion site^1^ |
| So_col1 | (miniISY100)**TA**CTAAACACTCAA | | SO_0146 | | ISSod3 transposase TnpA_ISSod3 | 155,208  (plus/minus) |
| So_col2 | (miniISY100)**TA**ATTCTAGCCCAA | | SO_2744 | | Helicase | 2,864,077  (plus/plus) |
| So_col3 | (miniISY100)**TA**CTTTAGTTTCAT | | SO_4478 | | Periplasmic stress-responsive two component signal transduction system histidine kinase CpxA | 4,666,745  (plus/minus) |
| So_col4 | (miniISY100)**TA**ACACACGTCTTG | | SO_2905 | | O-methyltransferase | 3,032,284  (plus/plus) |
| So_col5 | (miniISY100)**TA**ATATCAAAATCT | | SO_2188 | | Pseudogene ppkA | 2,297,961  (plus/plus) |
| *Pantoea ananatis* - NCBI: NC_017531.2 | | | | | | |
| Panan_col1 | | (miniISY100)**TA**ATCTGTTGGTATTG | Between PAJ_1800 and PAJ_1801 | | Intergenic region between genes *amsG* and *yegH* | 2,172,141  (plus/minus) |
| Panan_col2 | | (miniISY100)**TA**AAAGGTGTAACGTT | PAJ_3437 | | Polysaccharide biosynthesis protein | 4,123,495  (plus/minus) |
| Panan_col3 | | (miniISY100)**TA**GTTGCGGATCAGCC | PAJ_2303 | | Glutamate-cysteine ligase GshA | 2,791,080  (plus/plus) |
| Panan_col4 | | (miniISY100)**TA**TGAATGATTTGCTG | PAJ_3438 | | Glycosyltransferase | 4,124,595  (plus/minus) |
| Panan_col5 | | (miniISY100)**TA**ATTGTCATGAACTT | PAJ_1370 | | Osmotically inducible lipoprotein B precursor OsmB | 1,660,927  (plus/minus) |
| *Pseudomonas fluorescens* SBW25 - NCBI: NC_012660.1 | | | | | | |
| Pfluor_col1 | | (miniISY100)**TA**CCACGGATTTAGCT | Between PFLU_5988 and PFLU_5989 | Intergenic region between orotate phosphoribosyltransferase and putative exodeoxyribonuclease III | | 6,545,457  (plus/plus) |
| Pfluor_col2 | | (miniISY100)**TA**CAGCGCAAACCTCG | PFLU_3017 | Putative two-component system histidine kinase/response regulator fusion | | 3,285,953  (plus/plus) |
| Pfluor_col3 | | (miniISY100)**TA**CAACCTTCAGGCCG | PFLU_5061 | Nucleoside diphosphate kinase | | 5,562,076  (plus/plus) |
| Pfluor_col4 | | (miniISY100)**TA**CACAAGCAGTTACA | Between PFLU_3976 and PFLU_3977 | intergenic region between genes ECF-family sigma factor and putative periplasmic protein | | 4,391,646  (plus/minus) |
| Pfluor_col5 | | (miniISY100)**TA**TTTGTACCTAGAGG | PFLU_0823 | Dipeptide ABC transport system, substrate-binding protein | | 929,056  (plus/plus) |

^1^ the orientation of the transposon relative to the genomic sequence is indicated in brackets (plus/plus indicates that the coding strand of the kanR gene is in the same orientation as the reported genome sequence; plus/minus indicates they are in opposite directions).

Supplementary Table S2 – **Genome insertion sites of the miniISY100-LP transposon**

| *Escherichia coli* MG1655 – NCBI: CP027060.1 | | | | |
| --- | --- | --- | --- | --- |
| Mutant ID | Sequence | Locus tag | Gene product | Genome insertion site^1^ |
| MG1655_col1 | (miniISY100)  **TA**ATACTCAGGCAGTG | C5Y66_01305 | Phosphoribosylformylglycinamidine cyclo-ligase | 253,882  (plus/plus) |
| MG1655_col2 | (miniISY100)  **TA**ATGCGCGGGTAAAT | C5Y66_04440 | DedA family general envelope maintenance protein YqjA | 879,998  (plus/minus) |
| MG1655_col3 | (miniISY100)  **TA**AAACCAATCAATGA | C5Y66_04560 | Transcriptional regulator | 898,635  (plus/plus) |
| *Acinetobacter baumannii* ATCC19606 – BioCyc database | | | | |
| Abau_col1 | (miniISY100) **TA**TAATTATTTATTAG | / | PilB | supercont.1.5 39,439  (plus/minus) |
| Abau_col2 | (miniISY100) **TA**TGTACAAGTTAAAG | / | Hypothetical protein | supercont.1.1 9,676  (plus/plus) |
| Abau_col3 | (miniISY100) **TA**AAATCTGAGTTTTT | / | Threonine synthase / D-alanyl-D-alanine endopeptidase | supercont.1.3  392,142  (plus/plus) |
| *Acinetobacter baylyi* ADP1 – NCBI: CR543861.1 | | | | |
| Abay_col1 | (miniISY100) **TA**GTTAGAATCAAATG | ACIAD2890 | Conserved hypothetical protein; putative signal peptide | 2,830,424  (plus/plus) |
| Abay_col2 | (miniISY100)  **TA**TTTGCAATATCAAT | ACIAD2201 | Conserved hypothetical protein; putative membrane protein | 2,170,190  (plus/minus) |
| Abay_col3 | (miniISY100)  **TA**CTTTCCCAAGCTCA | ACIAD0702 | Putative transporter | 689,165  (plus/minus) |
| *Pseudomonas aeruginosa* PAO1 – NCBI: NC_002516.2 | | | | |
| Paer_col1 |  |  | Read stopped within transposon |  |
| Paer_col2 |  |  | Read stopped within transposon |  |
| Paer_col3 |  |  | Read stopped within transposon |  |

^1^ the orientation of the transposon relative to the genomic sequence is indicated in brackets (plus/plus indicates that the coding strand of the kanR gene is in the same orientation as the reported genome sequence; plus/minus indicates they are in opposite directions).
